# Efficacy of Pretreatment with Preservative-Free Topical Bromfenac in Improving Post-Intravitreal-Injection Pain: A Prospective Pilot Study

**DOI:** 10.3390/jcm11144172

**Published:** 2022-07-18

**Authors:** Dong-Hyun Lee, Minha Kim, Eun-Young Choi, Hee-Seung Chin, Min Kim

**Affiliations:** 1 Department of Ophthalmology, Inha University School of Medicine, Incheon 22332, Korea; wizmeca@live.co.kr (D.-H.L.); hschin@inha.ac.kr (H.-S.C.); 2 Inha Vision Science Laboratory, Inha University School of Medicine, Incheon 22332, Korea; 3 Department of Ophthalmology, Yonsei University College of Medicine, Seoul 03722, Korea; alsgk89@yuhs.ac (M.K.); eychoi@yuhs.ac (E.-Y.C.)

**Keywords:** nonsteroidal anti-inflammatory drug, analgesics, anti-VEGF treatment, bromfenac

## Abstract

(1) Background: To determine the analgesic effect of pretreatment topical bromfenac instillation in patients undergoing intravitreal anti-VEGF treatment. (2) Methods: A prospective, non-randomized pilot study was conducted in patients scheduled to receive repeated intravitreal anti-VEGF injections at a single tertiary hospital. Before the planned second injection, the patients received topical bromfenac eye drops twice a day for 3 days. At 1, 6, and 24 h after the first and second injections, the post-injection pain scores were determined using the numerical rating scale (NRS) telephonically. (3) Results: A total of 28 patients were enrolled in this study. After the first intravitreal injection, the NRS pain scores were 4.04 ± 1.90 at 1 h, 1.57 ± 1.75 at 6 h, and 0.93 ± 1.27 at 24 h. The pain scores after the second intravitreal injection significantly decreased at each measurement time point (*p* = 0.002, 0.055, and 0.004, respectively) compared to the first injection. (4) Conclusions: The use of topical bromfenac eye drops before intravitreal injection can lead to a significant improvement in injection-related pain scores, which is the basis for a future large-scale randomized comparative study.

## 1. Introduction

Intravitreal anti-vascular endothelial growth factor (anti-VEGF) injection therapy is widely used for the treatment of retinal vascular diseases. Since the therapeutic drug is directly injected into the eye, it acts quickly and is effective. However, as it is an invasive treatment, patients may experience pain or discomfort during or after the injection or may be afraid of the injection treatment. In addition, since multiple injections are often required, the pain and discomfort experienced by patients cannot be overlooked [1].

Pain that may occur during intravitreal injection therapy has been investigated and reported to be reduced with the concomitant use of topical nonsteroidal anti-inflammatory drugs (NSAIDs) [2,3,4]. NSAIDs result in pain improvement through the inhibition of the cyclooxygenase (COX) pathway and the production of prostaglandin [5,6]. Therefore, applications of topical NSAIDs in various ophthalmic surgeries have been attempted, typically to control pain and inflammation after cataract surgery and reduce the possible development of pseudophakic cystoid macular edema after cataract surgery.

Administration of nepafenac eye drops after intravitreal injection was shown to improve ocular discomfort [2]. In particular, bromfenac, a brominated ophthalmic NSAID, is known to have a lower half-maximal inhibitory concentration than other ophthalmic NSAIDs such as amfenac, ketorolac, and diclofenac in vitro. Therefore, it is approximately 3–4 times more potent in inhibiting COX-2 [7,8,9]. Moreover, bromfenac is known to penetrate the retinochoroidal tissue and shows excellent COX-2 inhibition and blood-retinal barrier breakdown [7]. Owing to these properties, bromfenac may be more advantageous than diclofenac or nepafenac for treating inflammatory diseases of the posterior segment.

Factors related to pain associated with intravitreal injection therapy include age, sex, and number of injections taken in the past. Older patients, women, and patients who have received several injections have low pain scores [10]. Existing studies have mainly used bromfenac eye drops in bottle formulations; therefore, evidence regarding the effect of preservative-free bromfenac in improving injection-related pain scores is insufficient. Preservative-free bromfenac has the advantages of the decreased risk of ocular surface damage and unstable tear film and similar or superior improvements in anterior chamber inflammation, dry eye parameters, and ocular and visual discomfort after cataract surgery, as seen with topical steroids [11].

This study aimed to analyze the efficacy of pretreatment use of preservative-free bromfenac eye drops in relieving pain after intravitreal anti-VEGF injection treatment.

## 2. Materials and Methods

This single-center, prospective, non-randomized, unblinded pilot study was conducted in accordance with the guidelines of the Declaration of Helsinki and approved by the Institutional Review Board of Severance Hospital (IRB approval number: 3-2019-0138). The inclusion criteria were as follows: (1) patients with planned intravitreal anti-VEGF injections (bevacizumab (Avastin, Genentech, South San Francisco, CA, USA), ranibizumab (Lucentis, Genentech, USA), or aflibercept (Eylea, Bayer, Pittsburgh, PA, USA)) at least 2 times and (2) patients more than 20 years old. The exclusion criteria were as follows: (1) patients with other eye diseases that may cause ocular pain (keratitis, glaucoma, ocular hypertension, endophthalmitis, uveitis, conjunctivitis, scleritis, and corneal epithelial defect); (2) patients who had difficulty in understanding and responding to the questionnaire because of severe cognitive, communication, and perception problems; and (3) patients aged below 20 years old.

In order to calculate the minimum number of subjects as a previous study for clinical trials, another paper on a similar subject was referred [2]. Using G Power (version 3.1), alpha = 0.05, power 80% was applied. A significant difference was determined when there was a difference of 0.5 points or more in pain scores between observations within the group.

After providing informed consent, all patients underwent history taking, corrected visual acuity and intraocular pressure measurement, slit-lamp and fundoscopic examination, and optical coherence tomography at the screening visit. The patients were scheduled for at least two consecutive intravitreal anti-VEGF injections, and telephonic questionnaires were used to quantify pain after 1 h, 6 h, and 24 h. Injection treatment was performed by the same doctor (D.H.L.). The injection treatment procedure was as follows: After instillation of 0.5% proparacaine hydrochloride (Alcaine, Alcon, Fort Worth, TX, USA) eye drops, the eyelids and conjunctival sac were sterilized and flushed with 5% povidone iodine. Then, the eyes were opened using the eyelid speculum. After instillation of 0.5% proparacaine hydrochloride (Alcaine, Alcon, USA) eye drops, 0.05 cc of anti-VEGF agent was injected into the inferotemporal or inferonasal area 3.5 mm posterior from the corneal limbus through a pars plana using a 30G needle. Topical moxifloxacin eyedrop (Vigamox, Alcon, USA) was instilled, and the eyelid speculum was removed. Since the injection treatment was performed on the same day as the patients’ outpatient visit, the injection timing of the patients was not the same. Before the planned second injection, the patients received topical bromfenac eye drops twice a day for 3 days. At the next visit, the examinations performed at the screening visit were repeated, and the pain scores after intravitreal anti-VEGF injection treatment were determined telephonically using the numerical rating scale (NRS). During the telephone questionnaire after the injection, the patients were asked whether they had redness, pain, decreased vision, or other discomfort, and additionally, whether other adverse reactions or symptoms occurred were checked at each outpatient visit.

Pain scores determined using the NRS were the primary outcome, and complications related to injection treatment or pretreatment topical bromfenac instillation were the secondary outcomes.

Statistical analyses were performed using SPSS Statistics v25.0 (IBM Corp., Armonk, NY, USA). Descriptive analysis was performed and the paired t-test and Wilcoxon’s rank-sum test were performed to analyze the change in pain scores after injection treatment. The pattern of changes in the pain scores after injection treatment was analyzed using a one-way repeated measures analysis of variance. A *p*-value of <0.05 was considered statistically significant.

## 3. Results

This section may be divided by subheadings. It should provide a concise and precise description of the experimental results, their interpretation, as well as the experimental conclusions that can be drawn.

### 3.1. Demographics of Patients

A total of 28 patients were enrolled, and their mean age was 68.1 ± 9.6 years. Of them, 12 (42.9%) were men. Twelve patients had a history of hypertension and diabetes, and 11 had a history of diabetes. The diagnoses of the patients were as follows: age-related macular degeneration (AMD) in 21 patients (75.0%), diabetic macular edema (DME) in five patients (17.8%), AMD with DME in one patient (3.6%), and macular edema secondary to branch retinal vein occlusion in one patient (3.6%). Patients were followed up for an average of 82.7 ± 77.9 days. The drugs used for treatment were as follows: aflibercept (Eylea^®^, Bayer, USA) in 15 patients (53.6%), bevacizumab (Avastin^®^, Genentech, USA) in 10 patients (35.7%), and ranibizumab (Lucentis^®^, Genentech, USA) in 3 patients (10.7%). There were 2 treatment naïve patients, and the remaining 26 patients received an average of 10.8 ± 10.4 injections (Table 1).

### 3.2. Change in Post-Injection Pain Scores after First and Second Injection Treatments

**Figure 1:**
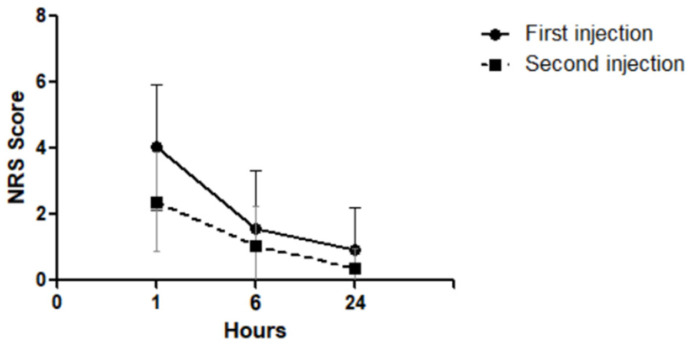
Change in post-injection pain scores after first and second injection treatments.

The change in pain scores was analyzed at different time points after the injection treatment. The pain scores after injection treatment determined telephonically showed a significant decrease over time (*p* < 0.001). After the pretreatment use of topical bromfenac eye drops for 3 days, the pain scores determined telephonically showed a significant decrease over time (*p* < 0.001). A comparison of the pain scores between the first injection treatment without bromfenac eye drops pretreatment and second injection treatments with bromfenac eye drops pretreatment showed that the pain scores decreased significantly after 1 and 24 h (*p* = 0.002 and 0.004, respectively). The pain score 6 h after injection treatment was lower after the first injection treatment than after the second injection treatment, but the difference was not statistically significant (*p* = 0.055, Table 2 and Figure 1).

During the study period, no patients required additional visits or treatment for complications related to the intravitreal injection treatment.

## 4. Discussion

The results of this study showed that after administering bromfenac eye drops before intravitreal injection treatment, the pain scores after intravitreal injection treatment improved significantly. No adverse events associated with the use of bromfenac eye drops before intravitreal injection therapy were observed.

Several studies have assessed the pain that may occur during or after intravitreal injection therapy. Ulrich et al. [2] found that the pain decreased significantly after topical nepafenac instillation after intravitreal injection. Georgakopoulos et al. [10] observed that the McGill pain questionnaire score significantly improved in the patient group in which bromfenac eye drops were instilled before intravitreal injection treatment than the control group. According to a meta-analysis published by Popovic et al., the pain score significantly decreased at 1, 6, and 24 h after topical NSAID instillation [3]. In terms of pain relief, the administration of bromfenac eye drops before the intravitreal injection is better than that after the intravitreal injection. In this study, after the instillation of bromfenac eye drops from three days before second intravitreal injection treatment, a significant improvement in the NRS pain score after injection treatment was confirmed compared to first injection treatment.

Pain scores after the topical bromfenac instillation were lower in old patients, female patients, and patients who had received multiple intravitreal injections than young patients, male patients, and patients who had received no or single intravitreal injections [10]. In this study, we could not clearly determine the factors related to pain after intravitreal injection because of the relatively small study population. However, considering that the average number of injections for the patients participating in this study was 10.8 ± 10.4, it can be considered helpful in relieving pain in patients who have had many injections in the past. Post-injection pain-related factors could be identified through a prospective study with a larger number of patients in the future.

In particular, using topical bromfenac before intravitreal injection can reduce patient anxiety [12]. Although this study did not quantify the patient anxiety, compliance with intravitreal injection treatment can also be evaluated if patient anxiety is quantified and analyzed in a future prospective study.

Complications that may occur with the use of topical bromfenac eye drops include ocular irritation, though serious complications such as corneal epithelial issues occur rarely [13]. In this study, no adverse events were observed with bromfenac use. Thus, in patients scheduled for repeated injection therapy, topical bromfenac instillation can be considered safe and used in the long term.

This study has several limitations. First, this study was conducted with a relatively small number of patients, and the time interval between the first and second injections was not consistent. Another limitation is that injection drugs and causative diseases are heterogeneous, which may have affected the pain reduction effect of bromfenac. In addition, there is no control group that did not use bromfenac eye drops in this study. Since the patients were informed that the pain relief effect after using bromfenac eye drops was to be analyzed, it is possible that the pain score after the second injection treatment was lower due to the expectation of the pain relief effect of the patients [14]. In a future study, subdividing and evaluating patients’ pain related to injection treatment into discomfort, itching, burning, and pain could also be a good way to analyze the effects of preservative-free drugs [15]. However, this is the first prospective study to prove the effect of preservative-free bromfenac eye drops on improvement in pain scores after intravitreal injection treatment and can serve as a basis for the design of a large-scale randomized comparative study.

## 5. Conclusions

In conclusion, the pretreatment use of preservative-free bromfenac eye drops in patients undergoing intravitreal injection therapy can significantly improve the post-injection pain scores and patient compliance with intravitreal injection therapy. In addition, this study can serve as a basis for the design of a large-scale randomized comparative study.

## Figures and Tables

**Table 1:** Demographics of patients.

	Number/Mean ± Standard Deviation
Patients (eyes)	28 (28)
Age (years)	68.1 ± 9.6
Sex (male:female)	12:16
Past history	
Hypertension	12
Diabetes	11
Diagnosis	
AMD	21 (75.0%)
DME	5 (17.8%)
AMD combined with DME	1 (3.6%)
Macular edema secondary to BRVO	1 (3.6%)
Follow-up period (days)	82.7 ± 77.9
Injected drugs	
Aflibercept	15 (53.6%)
Bevacizumab	10 (35.7%)
Ranibizumab	3 (10.7%)
Number of past injections	10.8 ± 10.4

AMD, age-related macular degeneration; DME, diabetic macular edema; BRVO, branch retinal vein occlusion.

**Table 2:** Change in post-injection pain scores after first and second injection treatments.

Post-Injection	NRS Score (After 1st Injection without Bromfenac Pretreatment)	NRS Score (After 2nd Injection with Bromfenac Pretreatment)	*p*-Value
1 h	4.04 ± 1.90	2.38 ± 1.50	0.002 *
6 h	1.57 ± 1.75	1.05 ± 1.20	0.055 ^†^
24 h	0.93 ± 1.27	0.38 ± 0.59	0.004 ^†^

* Paired t-test. ^†^ Wilcoxon signed rank test. NRS, numerical rating scale.

## Data Availability

The datasets generated during and/or analyzed during the current study are available from the corresponding author on reasonable request.
